# The Importance of Frontal QRS-T Angle in Predicting the Effectiveness and Success of Thrombolytic Therapy in Patients With Acute Pulmonary Embolism

**DOI:** 10.7759/cureus.33268

**Published:** 2023-01-02

**Authors:** Mehmet Durgun, Mehmet Zülkif Karahan

**Affiliations:** 1 Pulmonology, Dağkapı State Hospital, Diyarbakır, TUR; 2 Cardiology, Mardin Artuklu University Faculty of Medicine, Mardin, TUR

**Keywords:** hemodynamic instability, frontal qrs-t angle, echocardiography, thrombolysis, acute pulmonary embolism

## Abstract

Objective: The frontal QRS-T angle (fQRS-T) is associated with myocardial ischemia and ventricular arrhythmias. On the other hand, acute pulmonary embolism (APE) is a major risk factor for cardiac adverse events. This research aimed to determine whether the fQRS-T, a marker of ventricular heterogeneity, can be used to predict successful thrombolytic therapy in patients with APE.

Methods: This was a retrospective observational study. Patients diagnosed with APE and hospitalized in the intensive care unit between 2020 and 2022 were included in the research. A total of 136 individuals with APEs were enrolled in this research. The patients were divided into two groups: thrombolytic-treated (n=64) and non-treated (moderate to severe risk, n=72). An ECG was conducted for each patient, and echocardiography was performed.

Results: The mean age of the thrombolytic group was 58.2±17.6 years, with 35 females (55.1% of the group) and 29 males (44.9%). The non-thrombolytic group had a mean age of 63.1±16.2, with 41 females (56.5%) and 31 males (43.5%). Respiratory rate, heart rate, and fQRS-T were higher in the thrombolytic group, and oxygen saturation ratio and systolic and diastolic blood pressure were higher in the non-thrombolytic group (p=0.006, p<0.001, p=0.021; p<0.001, p=0.015, p<0.001, respectively). In the thrombolytic therapy group, comparing pre- and post-treatment ECG data revealed a statistically significant change in the fQRS-T value (p=0.019).

Conclusion: The fQRS-T may provide important clues for the successful treatment of APEs.

## Introduction

Acute pulmonary embolism (APE) is a life-threatening cardiovascular disease worldwide [1]. The incidence increases with advancing age [2]. Since it causes occlusion in the pulmonary vascular bed and causes mortality with right ventricular (RV) overload, diagnosis and treatment require urgency [3].

Dilatation in the ventricles due to afterload causes the remodeling of myocytes [4]. The resulting remodeling affects the cardiac conduction system and changes the cardiac axis. The QRS-T angle in the frontal plane (fQRS-T), which is the difference between the QRS and T axes, is a new indicator of ventricular repolarization diversity [5]. The increased fQRS-T score has been linked to adverse cardiac events [6].

In normotensive patients, anticoagulant therapy prevents thrombus growth and pulmonary embolism (PE) recurrence. In patients with hemodynamic instability, thrombolytic therapy should be administered to reduce PE-related mortality unless contraindicated [7,8]. Successful thrombolytic treatment has been described using transthoracic echocardiography (TTE) measurements, but there is no sufficient data regarding the relationship between ECG parameters and thrombolytic therapy.

This research aimed to reveal the effect of thrombolytic therapy on the fQRS-T and to determine its association with successful thrombolytic therapy.

## Materials and methods

Study design and subject

This retrospective investigation was performed in a tertiary hospital. Patients diagnosed with APE and who were admitted to the intensive care unit between January 2020 and June 2022 were included in the research. A total of 136 patients with APE were enrolled in this research. Patients with submassive or massive PE were treated with thrombolytics. The patients were divided into two groups: thrombolytic-treated (n=64) and non-treated patients (moderate to severe risk, n=72). Patients with coronary artery disorders, active cancer, hyperthyroidism, mild or severe valve disease, heart failure, chronic renal failure, left ventricular hypertrophy, chronic obstructive pulmonary disease, or congenital heart disease were excluded. Patients whose data were inaccessible and whose analysis was unsuccessful were excluded from this study. The local ethics committee (Gazi Yaşargil Training and Research Hospital) approved the study protocol (No: 2022-131). It adhered to the Declaration of Helsinki's ethical guidelines for human experimentation (date: 22/07/2022) (2013).

Study protocol

A 12-lead ECG was obtained using an electrocardiograph (model ECG-1350K, Nihon-Kohden Corporation) at a rate of 25 mm/s and an amplitude of 10 mm/mV. To digitize the existing ECGs, a scanner was used. At 400x magnification, two cardiologists calculated and analyzed the TpTe time. A significant and practically total agreement was observed between the analyses of the two cardiologists (κ=0.861). The QT interval and TpTe time for heart rate were adjusted using the Bazett formula. During ECG analysis, the QT interval, corrected QT (QTc) interval, QRS, and T axis were automatically identified and recorded. TTE was performed according to the American Society of Echocardiology [9]. The examinations were performed using a Philips ultrasonography device (EPIQ 7). Alteplase was administered as a thrombolytic agent. Alteplase (patients 65 kg or more) was given a 10 mg intravenous (IV) bolus, followed by a 90 mg IV infusion over 2 hours. In patients weighing less than 65 kg, the total dose was adjusted not to exceed 1.5 mg/kg. On average, a total of 10 mg IV bolus and 90 mg IV infusion doses (over 2 hours) were given. In combination with thrombolytics, a loading dose of 80 U/kg unfractionated heparin was administered intravenously, followed by an activated partial thromboplastin time (APTT)-adjusted infusion of 18 U/kg/h according to the guidelines [10].

Definitions

**Figure 1:**
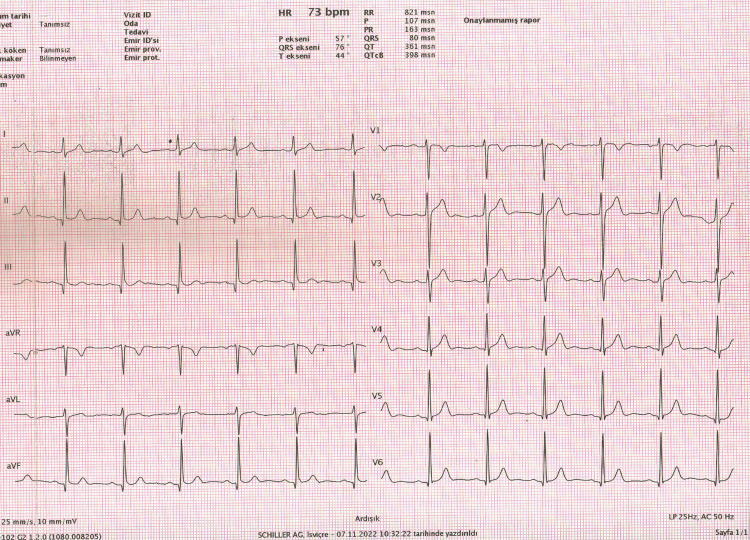
Calculation of frontal QRS-T angle from automated surface ECG report Frontal plane QRS-T angle (fQRS-T)=QRS axis-T axis (QRS°-T°)=76°-44°=32°

The fQRS-T is the angle formed by the difference between the QRS and T axes (Figure 1).

When the angle surpassed 180 degrees, it was changed to the lowest possible angle (360 degrees). The fQRS-T was generated from automatically obtained ECG data.

The P dispersion (Pd) was calculated by subtracting the shortest P wave length from the longest P wave duration. The QT and QTc distributions were determined in the same method [11].

Massive PE is defined as acute PE with obstructive shock or systolic blood pressure (SBP) <90 mmHg. Submassive PE is acute PE without systemic hypotension (SBP≥90 mm Hg) but with either right ventricular (RV) dysfunction or myocardial necrosis [12]. 

Statistics

IBM SPSS software (version 24.0; IBM Corp, Armonk, NY) was used for all the analyses. Initial continuous variables are shown as mean standard deviation or median (interquartile range). Applying Kolmogorov-Smirnov and Shapiro-Wilk tests, the normality distribution of the variables was determined. Categorical variables were represented with frequencies and percentages. Chi-square or Fisher's exact test was utilized for categorical variables. The Student's t-test or Mann-Whitney U-test was used to compare continuous variables. If the p-value was less than 0.05, tests were judged statistically meaningful.

## Results

The mean age of the thrombolytic group was 58.2±17.6 years, with 35 females (55.1% of the group) and 29 males (44.9%). The non-thrombolytic group had a mean age of 63.1±16.2, with 41 females (56.5%) and 31 males (43.5%). The baseline of the patients is summarized in Table 1.

**Table 1:** Baseline characteristics of the patients Data are expressed as mean SD, number (percentage), or median (interquartile range) as appropriate. DL: dyslipidemia; SBP: systolic blood pressure; DBP: diastolic blood pressure; fQRS-T: frontal plane QRS-T angle.

	Thrombolytic group N=64	Non-thrombolytic group N=72	p-Value
Age (years)	58.2±17.6	63.1±16.2	0.131
Gender, female, n (%)	35 (55.1)	41 (56.5)	0.712
Hypertension, n (%)	10 (16.4)	12 (16.8)	0.866
Diabetes mellitus, n (%)	5 (8.3)	7 (9.8)	0.628
DL, n (%)	8 (12.7)	9 (12.8)	0.910
Smoking, n (%)	10 (16.8)	11 (15.7)	0.821
Temperature (°C)	36.3±0.6	36.3±0.3	0.869
SatO_2_ (%)	82.65±7.03	86.69±4.82	<0.001
Respiratory rate	26.4±10.92	22±2.83	0.006
SBP (mmHg)	104.7±12.6	112.5±11.2	0.015
DBP (mmHg)	65.6±8.7	71.6±6.0	<0.001
Heart rate (bpm)	111.3±10.8	101.2±13.2	<0.001
fQRS-T (^0^)	62.4±35.6	48.3±31.1	0.021

Respiratory rate, heart rate, and fQRS-T were higher in the thrombolytic group; oxygen saturation ratio and SBP and diastolic blood pressure were higher in the non-thrombolytic group (p=0.006, p<0.001, p=0.021; p<0.001, p=0.015, p<0.001, respectively). The RV TTE measurements demonstrated worsened RV functions in the thrombolytic group (p<0.05, Table 2).

**Table 2:** Echocardiographic measurements of the groups Data are expressed as mean SD, number (percentage), or median (interquartile range) as appropriate. LVEF: left ventricular ejection fraction; LAD: left atrial diameter; RA: right atrium; TAPSE: tricuspid annular plane systolic excursion; RVGLS: right ventricular global longitudinal strain; PASP: pulmonary artery systolic pressure.

	Thrombolytic group N=64	Non-thrombolytic group N=72	p-Value
LVEF (%)	52.1±5.6	55.3±6.2	0.568
LAD (mm)	39.2±3.8	40.2±3.6	0.783
RA major diameter (mm)	48.1±8.4	40.3±9.2	<0.001
RA minor diameter (mm)	37.4±7.3	30.2±5.2	<0.001
TAPSE (mm)	16.5±1.3	18.8±3.2	<0.001
RVGLS (%)	-11.2±2.3	-15.3±2.8	<0.05
PASP (mmHg)	55.1±12.9	39.2±15.1	<0.001

Heart rate and fQRS-T demonstrated statistically significant group differences (p<0.05). Patients' electrocardiographic parameters were analyzed and evaluated (Table 3).

**Table 3:** Comparisons of ECG parameters before and after treatment Data are expressed as mean ± SD and median (interquartile range). Pd: P-wave dispersion; QTd: QT dispersion; QTcd: corrected QT dispersion; TpTe: T-peak to T-end; TpTec: corrected TpTe.

	Thrombolytic group N=64			Non-thrombolytic group N=72		
	Before	After	p-Value	Before	After	p-Value
Heart rate (bpm)	111.3±10.8	94.7±12.7	<0.001	101.2±13.2	93.4±11.6	0.108
Pd (ms)	51.1±9.1	48.3±13.6	0.329	47.2±11.4	46.8±9.2	0.508
QTd (ms)	54.0±6.6	51.3±8.1	0.204	52.8±9.5	51.6±6.7	0.412
QTcd (ms)	68.6±8.1	66.0±8.8	0.214	66.8±6.4	65.9±7.1	0.387
TpTe/QT	0.2±0.04	0.2±0.02	0.786	0.2±0.06	0.2±0.03	0.674
TpTe/QTc	0.19±0.05	0.19±0.33	0.954	0.18±0.12	0.18±0.07	0.908
fQRS-T (^0^)	62.4±18.6	48.8±16.7	0.019	48.3±21.5	45.9±20.9	0.387

In the thrombolytic therapy group, comparing pre- and post-treatment ECG data revealed a statistically significant change in the fQRS-T value (p=0.019).

## Discussion

This research revealed that the fQRS-T, which increased as a result of ventricular loading due to APE, decreased both clinically and statistically after successful thrombolytic therapy.

Thromboembolism causes an increasing afterload after dilatation in the right ventricle. The echocardiographic findings in our study support right ventricle dilatation in the thrombolytic group. Higher oxygen consumption is caused by shear stress in the ventricular wall, neurohormonal activation, and tachycardia [13]. Hypoxemia and hypotension may result in mortality [14]. There is neither a specific ECG finding to diagnose acute PE nor an ECG finding describing the effect and success of thrombolytic therapy. However, it can be expected that the change in the heart axis due to the enlargement of the right ventricle will be reflected in the ventricular repolarization parameters in the ECG recording.

In clinical practice, discovering novel ECG characteristics, such as fQRS-T, may be valuable for defining massive and submassive PE and predicting the effect and success of thrombolytic treatment in patients with APE. The fQRS-T, an indication of heterogeneity in ventricular activation and conduction, is the angle between ventricular depolarization and repolarization [15]. There are two methods for calculating the QRS-T angle. These are known as the spatial and fQRS-T, respectively. Calculating the spatial QRS-T angle is very difficult and requires advanced computer techniques [16]. In contrast, fQRS-T can be easily measured from the automatic report section of the ECG equipment and well matches the spatial QRS-T angle for risk calculation. Although there is no traditional upper limit for fQRS-T intervals, this value is often reported between 30 and 45 degrees [17]. Over the past decade, several observational studies have connected the QRS-T angle with sudden death and other fatal and pathological results [18,19]. Compared to electrocardiographic risk factors such as cQTd, a higher fQRS-T angle is recognized as a substantial and independent risk factor for adverse cardiac events [20].

In a study, fQRS-T was shown to be a predictor for mortality in patients with acute coronary syndrome [21]. Gül et al. reported that the prognostic value of the fQRS-T in individuals with non-ST-elevation myocardial infarction (NSTMI) and atrial arrhythmia was 81 degrees [22]. Colluoglu et al. stated that the fQRS-T was higher in ST-elevation myocardial infarction (STEMI) patients with unsuccessful fibrinolytic therapy than in successful therapy [23]. Zehir et al. showed that individuals with sluggish coronary flow and fQRS-T >93 degrees were more likely to have arrhythmic events [24]. The fQRS-T estimates mortality in heart failure patients, as reported by Gotsman et al. [25]. Usalp et al. highlighted that the fQRS-T was raised in subclinical hypothyroidism individuals who developed arrhythmia [26]. On the other hand, Günlü et al. revealed that the fQRS-T was not associated with arrhythmia in individuals with vertigo, brucellosis, and fibromyalgia [27-30].

Limitations

The population of the study was small. In this study, the estimated fQRS-T values ​​for both groups were observed to be lower than those previously reported in studies on cardiovascular disorders. This difference is due to the exclusion of participants with severe cardiovascular disease. The absence of a significant change in the frontal QRS-T angle in the non-thrombolytic patient group in the intensive care unit may be explained by the negligible effects of PE on the right ventricle or by the fact that the therapy had no impact on the right ventricle.

## Conclusions

This research indicated that the fQRS-T is an important indicator of ventricular loading in patients with APE. However, the non-invasive and comprehensible fQRS-T method in clinical follow-ups may provide important clues for the successful treatment of APE. Adverse events could be prevented by using this novel marker.
